# A Rare Presentation of Acute Respiratory Distress Due to Diffuse Large B-Cell Lymphoma of the Tongue Base

**DOI:** 10.7759/cureus.15124

**Published:** 2021-05-19

**Authors:** Muhammad Osto, Rafey Rehman, Alvin Ko

**Affiliations:** 1 Otolaryngology - Head and Neck Surgery, Wayne State University School of Medicine, Detroit, USA; 2 Department of Radiation Oncology, Beaumont Hospital, Royal Oak, USA; 3 Otolaryngology - Head and Neck Surgery, Henry Ford Health System, Detroit, USA

**Keywords:** non-hodgkin’s lymphomas, base of the tongue, respiratory distress, diffuse large b lymphoma, extranodal lymphomas

## Abstract

Primary diffuse large B-cell lymphoma of the tongue base (BOT) is an extremely rare entity with only a few cases described in the English literature to date. The incidence of BOT non-Hodgkin’s lymphoma (NHL) increases with age, most commonly after the sixth decade of life with no observed gender differences. Our patient presented with a six-month history of right neck swelling, one-month history of dysphagia, a change in voice, and ultimately acute airway distress, which led to a tracheostomy. We report an extremely rare case of a diffuse large B-cell lymphoma presenting with airway distress. The patient was treated using rituximab-cyclophosphamide-doxorubicin-vincristine-prednisone (R-CHOP) chemotherapy, a five-day steroid course, and one intrathecal methotrexate. The patient recovered completely and is alive at the time of this writing. NHLs occur more commonly in patients like ours with a prior history of congenital immunodeficiency and celiac disease, exposure to radiation, acquired immune deficiency syndrome, rheumatoid arthritis, or Sjögren’s syndrome. Most reported cases of BOT NHLs may cause dysphagia, pharyngeal foreign body sensation, or progressive dyspnea. This case highlights that although NHL of the tongue is a very rare entity, it should not be overlooked and should always be in the differential diagnosis among various benign and malignant tumors and may cause rapid respiratory deterioration.

## Introduction

Lymphomas are malignant neoplasms arising from the lymphocyte cell lines in the lymph nodes, usually affecting other lymphoid organs like the spleen. Lymphomas present with a wide spectrum of clinical and pathological features and are classified as either Hodgkin’s or non-Hodgkin’s lymphoma (NHL). Extranodal lymphomas are a rare presentation with only 3%-5% of malignant lymphomas of the head and neck region arising in the oral cavity [1]. Following squamous cell carcinomas and salivary gland neoplasms, lymphomas are the third most common tumor of the oral cavity. However, primary oropharynx lymphomas account for only 1% of all lymphomas with most arising in the Waldeyer’s ring [2,3]. Isolated primary NHL of the tongue base is an extremely rare entity among lymphomas of the oropharynx. The most common presenting complaints of lymphoma of the tongue base (BOT) include asymptomatic incidental findings, globus sensation in the throat, dysphagia to solid foods, or odynophagia. We report a case of a patient with diffuse large B-cell lymphoma (DLBCL) of the base of the tongue with a six-month history of neck swelling, a one-month history of dysphagia, voice change, and ultimately acute airway distress requiring tracheostomy.

## Case presentation

**Figure 1:**
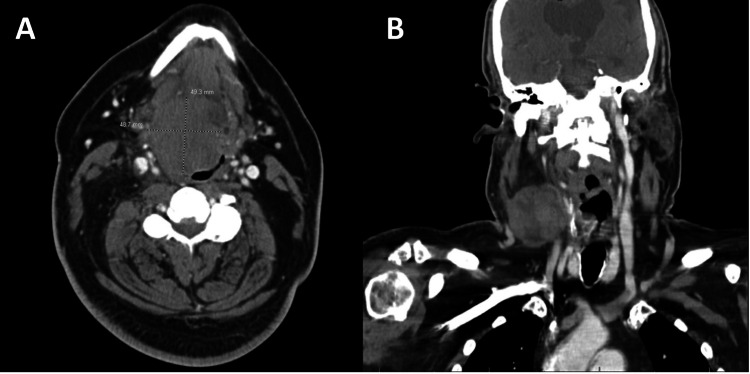
Contrast-enhanced computed tomography (CT) of the neck (A) Large right tongue base mass largely obliterating the oropharyngeal airway. (B) Prominent necrotic lymph node noted in the right level II/III of the neck.

A 62-year-old male was transferred from a neighboring hospital with airway distress after a six-month history of right neck swelling, a one-month history of dysphonia, dyspnea when supine, and dysphagia. He denied fevers, chills, night sweats, or weight loss. His medical history included IgA deficiency, epilepsy, and celiac disease. He did not smoke, chew tobacco, or consume alcohol. He was HIV-negative with no personal or family history of malignancy or radiation exposure. On examination, the patient had a closed-sounding voice, no drooling or stridor, and a 4-cm firm, non-tender swelling in the right level II/III of the neck. Flexible nasopharyngolaryngoscopy revealed a prominent right tongue base mass obstructing the vallecula and pushing the epiglottis against the posterior pharyngeal wall and edematous right arytenoid mucosa. Contrast-enhanced computed tomography (CT) of the neck showed a 4.9 cm x 4.9 cm x 5.6 cm mass involving the base of tongue, oral cavity, hypopharynx, and preepiglottic fat with near-complete obliteration of the oropharyngeal airway. Additionally, a 3.3 cm x 4.2 cm x 6.1 cm partially necrotic lymph node was noted adjacent to the right hyoid (Figure 1).

**Figure 2:**
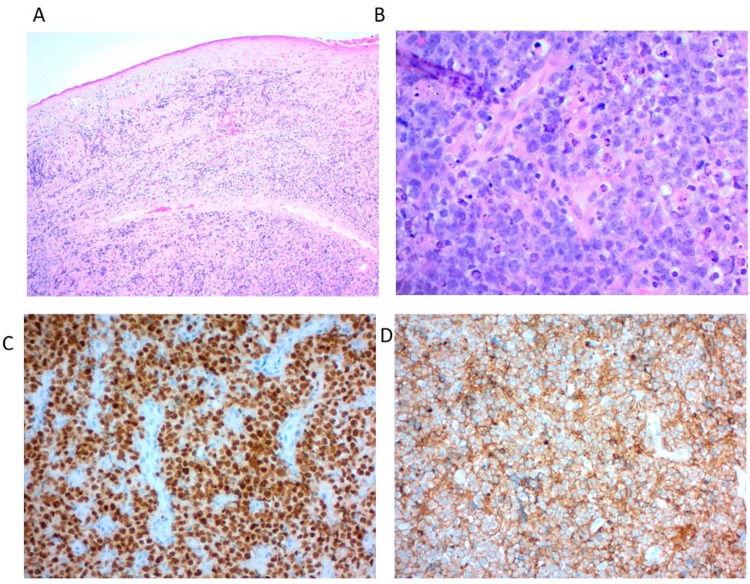
Histology (A) Diffuse sheets of atypical and large discohesive lymphoid cells under unremarkable squamous mucosa. (B) Atypical lymphoid cells with signs of apoptosis and mitosis. (C) The atypical lymphoid cells are positive for Pax5. (D) The cells are also positive for CD10.

While intravenous steroid administration in the ICU decreased his arytenoid swelling, concern for impending airway distress remained high, given his voice changes and the size of the mass. The patient ultimately agreed to awake tracheostomy followed by direct laryngoscopy and excisional biopsy. Pathology from the large exophytic mass of the tongue base showed squamous mucosa with diffuse sheets of atypical, large discohesive lymphoid cells that were Pax5- and CD10-positive and showing apoptosis and mitosis (Figure 2).

Fluorescence in situ hybridization showed no MYC, BCL2, and BCL6 gene rearrangements. Postoperative recovery was complicated by aspiration pneumonia from which he recovered with routine treatment. Positron emission tomography or CT showed no other sites of involvement, establishing a final diagnosis of stage II diffuse large B-cell germinal center-type lymphoma. The patient was started on rituximab-cyclophosphamide-doxorubicin-vincristine-prednisone (R-CHOP) chemotherapy as well as one course of intrathecal methotrexate. Treatment was complicated by an episode of neutropenic sepsis after two cycles of R-CHOP, after which he recovered and is now currently finished with his third cycle of R-CHOP.

## Discussion

Primary DLBCL of the tongue base is a rare entity with only 14 cases described in the English literature to date. The incidence of BOT NHLs increases with age, most commonly after the sixth decade of life with no observed gender differences [4]. The most common head and neck site for extranodal malignant lymphomas involves Waldeyer’s ring, an annular area of lymphoid tissue from the nasopharynx down each lateral pharyngeal wall to the palatine tonsils before meeting again at the base of the tongue. Other less common NHL sites include the nose and paranasal sinuses, orbit, and the salivary glands [1]. NHLs occur more commonly in patients with a prior history of congenital immunodeficiency and celiac disease, exposure to radiation, acquired immune deficiency syndrome, rheumatoid arthritis, or Sjögren’s syndrome [5]. Most reported cases of NHLs of the tongue base may cause dysphagia, pharyngeal foreign body sensation, or progressive dyspnea [4,6,7]. The patient presented with a six-month history of right neck swelling, one-month history of dysphagia, change in voice, and acute airway distress, which led to a tracheostomy. Only Singh et al. [6] reported a patient with a change in voice, and only Hmidi et al. [8] reported their case having upper airway obstruction.

Lymphomas cannot be reliably differentiated from other benign or malignant lesions such as squamous cell carcinoma (SCC) solely based on exam or imaging. SCC was felt to be the most likely initial diagnosis for our patient despite the lack of tobacco/nicotine and alcohol use history because of the overall increasing oropharyngeal cancer incidence attributed to increased human papillomavirus infection exposure [9]. Imaging such as CT or magnetic resonance imaging can help delineate NHL disease extent, but the diagnosis can only be confirmed by biopsy and immunohistochemical analysis [4]. Lingual thyroids are an uncommon congenital diagnosis, with papillary thyroid cancer arising in this site rarer still [10]. The lateral and superficial BOT lesion position and presence of a thyroid gland in a normal position argue against this diagnosis despite the signs of malignant lymphadenopathy, a common finding in papillary thyroid cancer. Polymorphous adenocarcinoma is a low-grade salivary gland malignancy with a propensity for regional lymphatic spread but is uncommonly found in the tongue base.

Airway concerns in the BOT subsite must be carefully considered primarily during initial presentations and secondarily as many treatment regimens may cause delayed airway distress from tumor edema and/or mucositis. Most of the head and neck NHLs are of B-cell origin, most commonly the diffuse large B-cell type. Due to its low incidence, no consensus or treatment guidelines exist for BOT lymphoma. Many NHLs show good sensitivity to typical chemotherapy regimens such as the R-CHOP regimen used in our patient [11]. Some have recommended treating well-localized lesions below stage II with radiotherapy alone or with excisional biopsy followed by radiotherapy [12]. Given the rarity of these tumors, it will prove challenging to conduct randomized controlled trials that can confirm the findings of this paper. Instead, an appropriately designed retrospective cohort study could provide meaningful guidance for the treatment of NHL of the BOT.

## Conclusions

In conclusion, we are reporting one of the few cases of extranodal primary NHL of the BOT presenting with acute airway distress in a patient with celiac disease and IgA deficiency. Although NHL of the tongue base is a very rare entity, it should not be overlooked and should always be in the differential diagnosis among various benign and malignant tumors. Diagnosis should be established with biopsy and immunohistochemical analysis as treatment and prognosis of malignant lymphomas are vastly different than other pathologies of this region.
